# Net buffer load during regional citrate anticoagulated continuous renal replacement therapy

**DOI:** 10.1371/journal.pone.0315727

**Published:** 2025-01-30

**Authors:** Innas Forsal, Dominique Pouchoulin, Viktoria Roos, Jorge Echeverri, Marcus Ewert Broman

**Affiliations:** 1 Baxter International Inc., Lund, Sweden; 2 Baxter International Inc., Meyzieu, France; 3 Baxter International Inc., Deerfield, Massachusetts, United States of America; 4 Skåne University Hospital, Lund, Sweden; Sant Anna Hospital: Clinica Sant’Anna, SWITZERLAND

## Abstract

**Background:**

Regionally anticoagulated continuous renal replacement therapy with citrate is the first choice for critically ill patients with acute kidney injury. If citrate that reaches the patient exceeds the metabolic capacity, metabolic alkalosis will follow. Bicarbonate from the treatment fluids will also reach the patient and add to the bicarbonate load. Net buffer load is a parameter calculated by the dialysis machine software from the treatment fluid contents, the fluid flows and the filter properties. Each time the treatment settings are altered the net buffer load will be re-calculated. This parameter is integrated as a static safety parameter in the Prismax version 3 software, and will guide the operator on a possible development of metabolic alkalosis with the current settings.

**Methods:**

Algorithms for estimating the net buffer load were developed. Hourly clinical data from electronic medical records from 60 patients’ continuous renal replacement treatments at the Adult Intensive Care Unit, Skåne University Hospital, Lund, Sweden was used to simulate net buffer load. The relation between net buffer load and acid base status at steady state was evaluated. Net buffer load was also calculated from three previous studies for comparison to our present cohort.

**Results:**

The mean net buffer load was +0.09 ±0.04 mmol/h/kg in the present cohort, and comparable to historic cohorts from literature. We could not establish a correlation to steady state arterial bicarbonate. The pre blood pump citrate containing replacement fluid flow rate was affecting net buffer load most of all parts of the dialysis circuit, with a r^2^ Pearson correlation coefficient of 0.65 (p<0.001).

**Conclusion:**

The net buffer load parameter can alert the operator on the expected impact of the dialysis circuit on patient’s acid base status. It was possible to calculate realistic net buffer load values during 60 CRRT treatments.

## Introduction

Continuous renal replacement therapy (CRRT) is the first choice for critically ill patients with acute kidney injury (AKI) and simultaneous hemodynamic instability, recommended by the KDIGO guidelines [1]. Anticoagulation is needed during CRRT to prevent clotting when blood comes into contact with the tubing of the circuit and the filter. The clotting process will take place gradually and reduce the lifespan of the filter [2].

Regional citrate anticoagulation has proved to be superior to systemic anticoagulation, like heparin. Citrate leads to decreased risk of bleeding [3,4], increased filter life span, decreased rate of complications and decreased therapy interruptions [5]. Regional anticoagulation with citrate is more efficient and more safe [6].

Citrate is infused into the blood at the beginning of the extracorporeal circuit in order to provide anticoagulation by chelation of ionized (free) calcium (iCa). The citrate-calcium complexes are removed through the filter membrane due to their small molecular size. This leads to an ongoing loss of calcium over the filter membrane, and a matched substitution of calcium is needed in order to avoid systemic hypocalcemia in the patient. However, a fraction of the iCa-citrate complexes will reach the patient, and constitute a citrate load, which will be metabolized to bicarbonate mainly by the liver and skeletal muscles, by fitting into the Krebs (citric acid) cycle. Each citrate molecule yields three bicarbonate molecules [7].

Citrate toxicity can occur in two different forms: 1. excessive amount of citrate reaching the patient and being metabolized to bicarbonate leading to metabolic alkalosis and 2. the amount of citrate reaching the patient exceeds the metabolic capacity of citrate leading to direct citrate accumulation and adjacent metabolic acidosis. These two adverse effects can be effectively reduced by regular monitoring of laboratory values such as systemic iCa and by usage of structured protocols [8].

Systemic acid-base balance in a CRRT patient with regional citrate anticoagulation is impacted by 1. citrate load, 2. the patient’s citrate metabolism capacity, 3. bicarbonate from the treatment fluids, 4. other buffer sources, and of course 5. the underlying acid base disturbance from the disease state itself.

Dialysate and replacement solutions contain bicarbonate and depending on the concentrations and fluid rates, different amount of bicarbonate will enter the blood stream and must be accounted for in the total buffer load.

In this study, we introduce the normalized net buffer load (nNBL_25_) version 1, which is calculated from the CRRT fluid flow settings, the fluid content of citrate and bicarbonate and the properties of the filter. This parameter is introduced in PrisMax V3 (software version 3, Baxter, Deerfield, USA) and is an estimated, static parameter, which is re-calculated each time the treatment settings are altered in order to indicate that the impact of the new CRRT settings on the patient’s acid base status are neutral, or to alert that the new CRRT settings will have an acidifying impact or an alkalinizing impact systemically. nNBL_25_ can be used in any treatment mode, even without regional citrate anticoagulation. It can be visualized on the CRRT machine’s interface.

The aims of this study were to develop equations for calculating nNBL_25_ and to simulate the parameter in a retrospective cohort consisting of 60 critically ill patients who all received CRRT treatments during their stay in the ICU, and to compare nNBL_25_ to acid-base parameters including bicarbonate, pH and base excess (BE).

## Introducing net buffer load

The parameter net buffer load (NBL) is defined as the sum of 1. bicarbonate generated from the metabolism of citrate in the patient, and 2. bicarbonate balance between the patient and the CRRT circuit. NBL can be seen as the amount of buffer compensating the buffer deficit in the patient during CRRT. NBL can further be normalized to the actual patient weight (nNBL). The assumed venous bicarbonate concentration in the first version of the mathematical model was fixed to 25 mmol/L, which matches the desirable steady state, hence the annotation nNBL_25_.

It is important to realize that nNBL_25_ is a balance. A negative value means that the CRRT circuit is removing bicarbonate from the patient, who will go towards acidosis. A zero value means that the circuit will not infuse nor consume bicarbonate, whereas a positive balance means that the circuit will add bicarbonate and neutralize existing acidosis in the patient, who will experience an alkalinizing effect.

The software of the CRRT machine will calculate (estimate) the nNBL_25_ based on the content of the treatment fluids in use, filter membrane properties, and the amount of added citrate to the circuit. A fraction of the citrate will be dialyzed away as calcium-citrate complexes and another fraction will go into the patient’s blood and be metabolized to bicarbonate. The nNBL_25_ estimates how all this will happen for each set of treatment settings.

The nNBL_25_ is a marker for an add-on effect of the CRRT circuit on the patient, due to the current treatment settings.

It is important to remember that this effect comes on top of the already existing acid-base disturbance in the patient.

The nNBL_25_ is to be considered as an estimated static safety parameter of the current treatment prescription programmed in the CRRT machine. It is included on the interface and can alert the operator on the estimated result of the current machine settings. Each time the treatment settings are altered the nNBL_25_ will be re-calculated and get a new value.

### Equations

Net buffer load (NBL_25_) is determined according to Eq 1 as a function dependent on the concentration of bicarbonate in blood and bicarbonate precursors, which consist of bicarbonate generated from the citrate metabolism (*J*_*metcit*_). The citrate load is multiplied by three since metabolism of one citrate molecule generates 3 bicarbonate ions. In addition, the netto balance of bicarbonate defined by the difference between the bicarbonate content in the treatment fluids and the bicarbonate lost over the filter membrane to the effluent fluid (*J*_*HCO3bal*_ = *J*_*HCO3inf*_ − *J*_*HCO3eff*_) will count. Finally, the CRRT treatment settings, i.e. the fluid flows will also have an impact.


$$NBL_{25}=J_{metcit}+J_{HCO3bal}=3xJ_{citrateload}+(J_{HCO3inf}-J_{HCO3eff})$$ (1)

Citrate load is defined more in detail as the net infusion rate of citrate to the patient, calculated by the difference between the citrate infusion rate from citrate containing prefilter replacement solution (*J*_*citPBP*_) and the citrate removal rate to the effluent (*J*_*citeff*_). Citrate infusion rate is calculated as the product of the citrate dose and the blood flow, while citrate removal rate is calculated as the product of the citrate clearance (*K*_*cit*_) and the citrate concentration at filter inlet (*C*_*citinlet*_), according to Eq 2.


$$J_{citrateload}=J_{citPBP}-J_{citeff}=D_{cit}xQ_{b}-K_{cit}xC_{citinlet}$$ (2)

The bicarbonate balance is dependent on the infusion or loss of bicarbonate in the extracorporeal circuit, and thus calculated as a product of the infusion of bicarbonate (which equals dialysate and/or replacement fluids’ *(J*_*HCO3inf*_) bicarbonate content) and the bicarbonate removal rate over the filter membrane to the effluent (*J*_*HCO3eff*_), according to Eq 3.


$$\begin{array}{ll} J_{HCO3bal} & =J_{HCO3inf}-J_{HCO3eff}=\left(Q_{dial}xC_{HCO3dial}+Q_{rep}xC_{HCO3rep}\right)\\ & -(Q_{dial}xC_{HCO3dial}+K_{HCO3}x\left(C_{HCO3inlet}-C_{HCO3dial}\right)+Q_{fil}xC_{HCO3dial}) \end{array}$$ (3)

The infusion rate of bicarbonate is dependent on the dialysate fluid bicarbonate content and the dialysate flow rate (Q_dial_) and the replacement fluid bicarbonate content and the replacement fluid flow rate (Q_rep_).

The bicarbonate removal rate over the filter membrane to the effluent is dependent on the filter clearance rate of bicarbonate (*K*_*HCO3*_), the bicarbonate concentration at the filter inlet (*C*_*HCO3inlet*_), the dialysate bicarbonate concentration, and on removal of bicarbonate due to the ultrafiltration rate (*Q*_*fil*_).

The bicarbonate concentration at the filter inlet is a result of patient HCO_3_^-^ (assumed as 25 mmol/L in this version 1 model), and the predilution from the prefilter replacement solution.

The citrate concentration of the prefilter replacement solution is dependent on the prefilter replacement solution dilution.

### Assumptions and decisions

The following important assumptions were made: **1**. citrate metabolism is proportional to body weight, **2**. patient citrate concentration is computed at steady state, with a metabolic clearance of 700 mL/min, **3**. The computed nNBL_25_ does not match to the actual balance of running therapy, but instead to steady state, when patient venous bicarbonate is assumed to stabilize at 25 mmol/L, **4**. Steady state was assumed to be reached after 48 hours, **5**. lactate was ignored.

### Steady state

Reaching the steady state requires a time period, since the effect of bicarbonate alters the metabolic component of the acid base equilibrium [9]. Other reasons for the required time period until steady state are the high distribution volume of bicarbonate and the low intensity of the CRRT therapy.

## Methods

### Cohort, inclusion and exclusion criteria

All patients with critical illness and adjacent acute kidney injury requiring CRRT on clinical grounds, starting from year 2010 and onwards were screened in the electronic medical record (ICCA, Philips, Eindhoven, Netherlands) at the Adult Intensive Care Unit, Skåne University Hospital, Lund, Sweden. The inclusion criteria were: 1. complete CRRT data available for extensive high-resolution calculation of the nNBL_25_, 2. uninterrupted treatment for at least 72 hours from CRRT treatment start, 3. citrate as the only anticoagulation, and 4. available complete set of acid-base parameters throughout the CRRT treatment. The exclusion criteria were: 1. insufficient data, 2. the patient was included in other studies, 3. patient’s age <18 years, and 4. interruptions in the CRRT treatment defined by lacking data for at least one treatment hour.

### CRRT protocol

The Prismaflex machine and ST-150 filters were used in all patients. The modality was continuous veno-venous hemodiafiltration (CVVHDF). The treatment solutions used for all patients were Prismocitrate 18/0 (citrate containing prefilter replacement solution; citrate 18, Na 140, Cl 86, bicarbonate 0 mmol/L), Prism0cal B22 (calcium-free dialysate solution; Na 140, K 4, Cl 122.5, Mg 0.75, lactate 3, glucose 6.1, bicarbonate 22 mmol/L), and Phoxilium (post-filter replacement solution; Na 140, K 3.0, Cl 115.9, Ca 1.25, Mg 0.6, phosphate 1.2, bicarbonate 30 mmol/L). The Flexitrate protocol **(see supplement)** was utilized during the CRRT therapy for all patients. The Flexitrate treatment regime was rigorously maintained based on regular measured simultaneous systemic ionized calcium (normal range 1.0–1.2 mmol/L) and post-filter ionized calcium (normal range 0.25–0.50 mmol/L) targets throughout the treatments [1]. All treatment corrections were carried out according to the Flexitrate protocol and according to clinical decisions, and not based on the nNBL_25_ value.

### Extraction of data

Biochemistry, patient data, and Prismaflex treatment data were extracted from the ICCA system and sorted to match fluid flows according to the actual timestamp. All parameters in the medical records carry a value, a patient identification number and a timestamp. Treatment data from the Prismaflex CRRT machines were automatically uploaded to the medical records system during the treatments. A study period of the first 72 hours from the start of CRRT treatment was defined. All data was extracted from the medical records system to excel files and uploaded further to Matlab 2022b. The processing of data was carried out in Matlab 2022b and the calculation of nNBL_25_ was done by using a script created in Matlab 2022b. Outliers were present in the dataset for nNBL_25_ calculation and were; zero values for flows, and extreme or un-physiological acid base parameters.

### Ethical approval

The Regional Ethics Board of southern Sweden approved the study (Dnr 2017/618). Individual consent from the patients was waived. The only intervention was extraction of treatment data. All treatment decisions were made on clinical grounds by the physician in charge.

### Statistical analysis

Two-sample t-test was used to compare means of two independent groups. Significance level was set to p<0.05. The data was checked for normality and equal variances.

The t-test was conducted by using MATLAB’s ttest2 function.

### Outcome

To calculate the absolute nNBL_25_ level for each patient in the study cohort and to evaluate if the level correlated to steady state bicarbonate.

## Results

### Cohort

Of a total of 120 patients identified who got CRRT treatment during years 2010–2017 and screened for inclusion, 60 patients fulfilled the inclusion criteria and not the exclusion criteria, and were subsequently included in the final cohort. Patient characteristics of the cohort are presented in Table 1. 36/60 patients’ main diagnosis was septic shock, 6/60 patients had post-resuscitation critical care after cardiac arrest, and the remaining 18/60 patients had critical care after major surgery, cerebral catastrophes, intoxications, airway bleedings and rhabdomyolysis. All patients had multiorgan failure and acute kidney injury which required CRRT.

**Table 1:** Baseline characteristics of the patients in the study cohort.

	All patients	Survivors	Non-survivors	Unit	p-value survivors/non-survivors
**Age**	54 ±21	48 ±11	58 ±19	years	0.044*
**Women**	43.7	40.1	45.5	percent	0.078
**Men**	56.3	59.9	54.5	percent	0.089
**SAPS3**	75.7 ±14.9	72.3 ±12.6	88.5 ±14.7		0.003*
**SOFA**	11.2 ±3.7	10.1 ±2.9	15.8 ±3.5		<0.001*
**Mean arterial pressure**	51.1 ±9.3	52.0 ±9.5	47.3 ±7.5	mmHg	0.093
**Total bilirubin**	63.1 ±82.0	51.2 ±67.2	118.6 ±118.4	μmol/L	0.005*
**KDIGO2** **at treatment start**	60	65	55	percent	0.036*
**KDIGO3** **at treatment start**	40	35	45	percent	0.039*
**CRRT duration**	185 ±168	189.8 ±148	159.5 ±38	hours	0.004*
**Creatinine**	223 ±94	180 ±37	259 ±20	mmol/L	0.035*
**Urea**	18.4 ±7.3	16.3 ±6.5	20.6 ±4.9	mmol/L	0.043*
**Bicarbonate**	23.6 ±2.8	24 ±1.6	23 ±3.6	mmol/L	0.184
**Base excess**	-1 ±3.2	-0.5 ±1.7	-1.6 ±4	mmol/L	0.132
**Sodium**	138.0 ±3.0	139 ±2	134 ±4	mmol/L	0.154
**Potassium**	4 ±0.3	4.1 ±0.2	4 ±0.4	mmol/L	0.129
**Lactate**	1.8 ±1.4	1.7 ±1.2	1.9 ±1	mmol/L	0.157
**pH**	7.4 ±0.09	7.41 ±0.06	7.38 ±0.10		0.142

Means and standard deviations were calculated for all 60 included patients, the 35 survivors and the 25 non-survivors. Significant difference is marked with asterix *, by using criteria p<0.05.

35/60 patients survived the hospital stay (survivors regarding hospital mortality) and 25/60 patients died in the hospital (non-survivors). No patient died during the 72 hour study period.

It was possible to calculate realistic nNBL_25_ values throughout the CRRT treatments for all included patients in the cohort.

No direct citrate toxicity occurred during the investigated 60 CRRT treatments. The ratio total calcium/ionized calcium ratio was calculated daily and remained within normal range (<2.5) at all times. No severe cases of metabolic alkalosis (pH > 7.6) were observed.

### nNBL_25_ levels in the cohort

**Fig 1:**
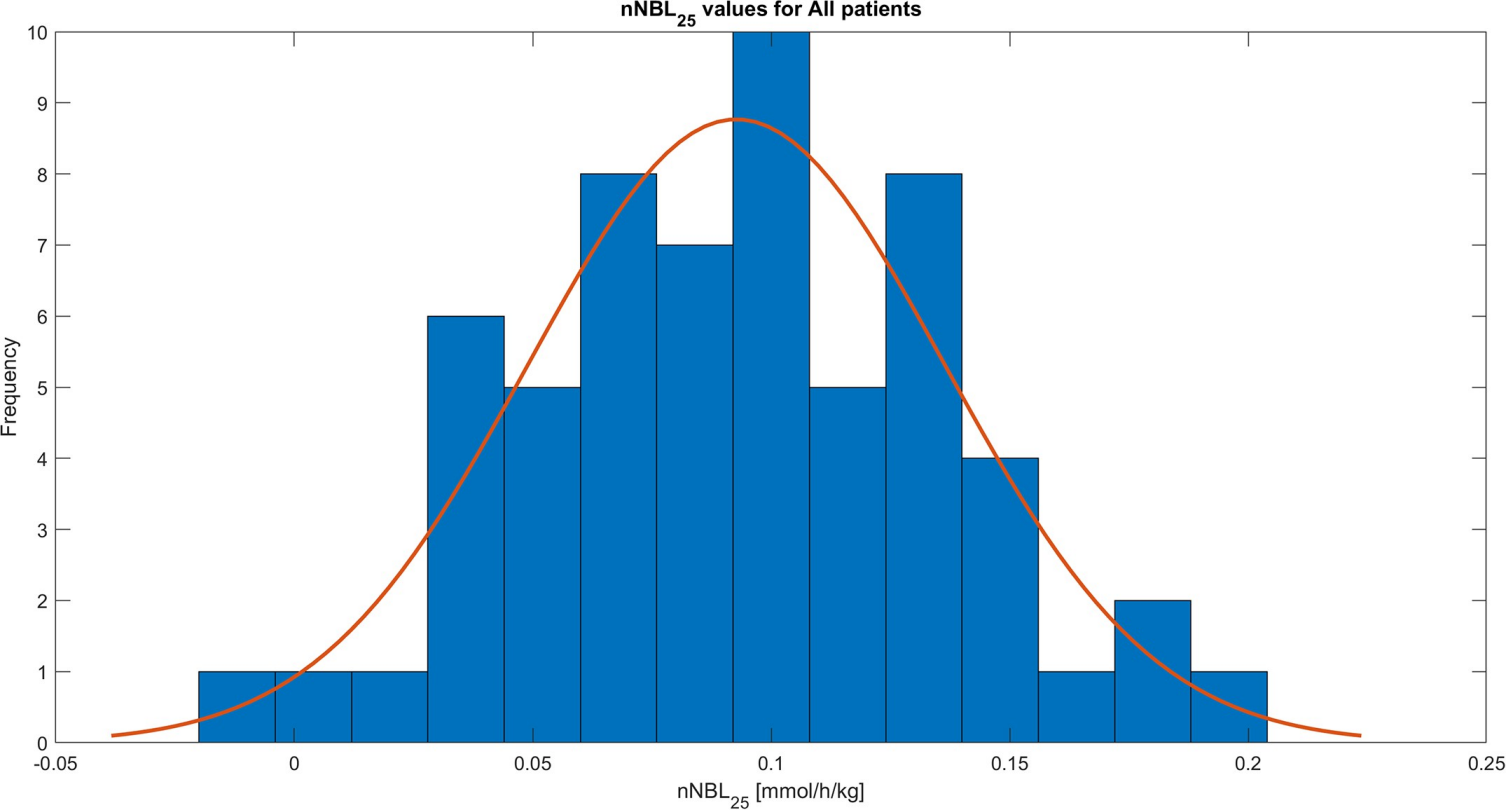
Histogram of the patient nNBL_25_ treatment means for all the 60 included patients.

**Fig 2:**
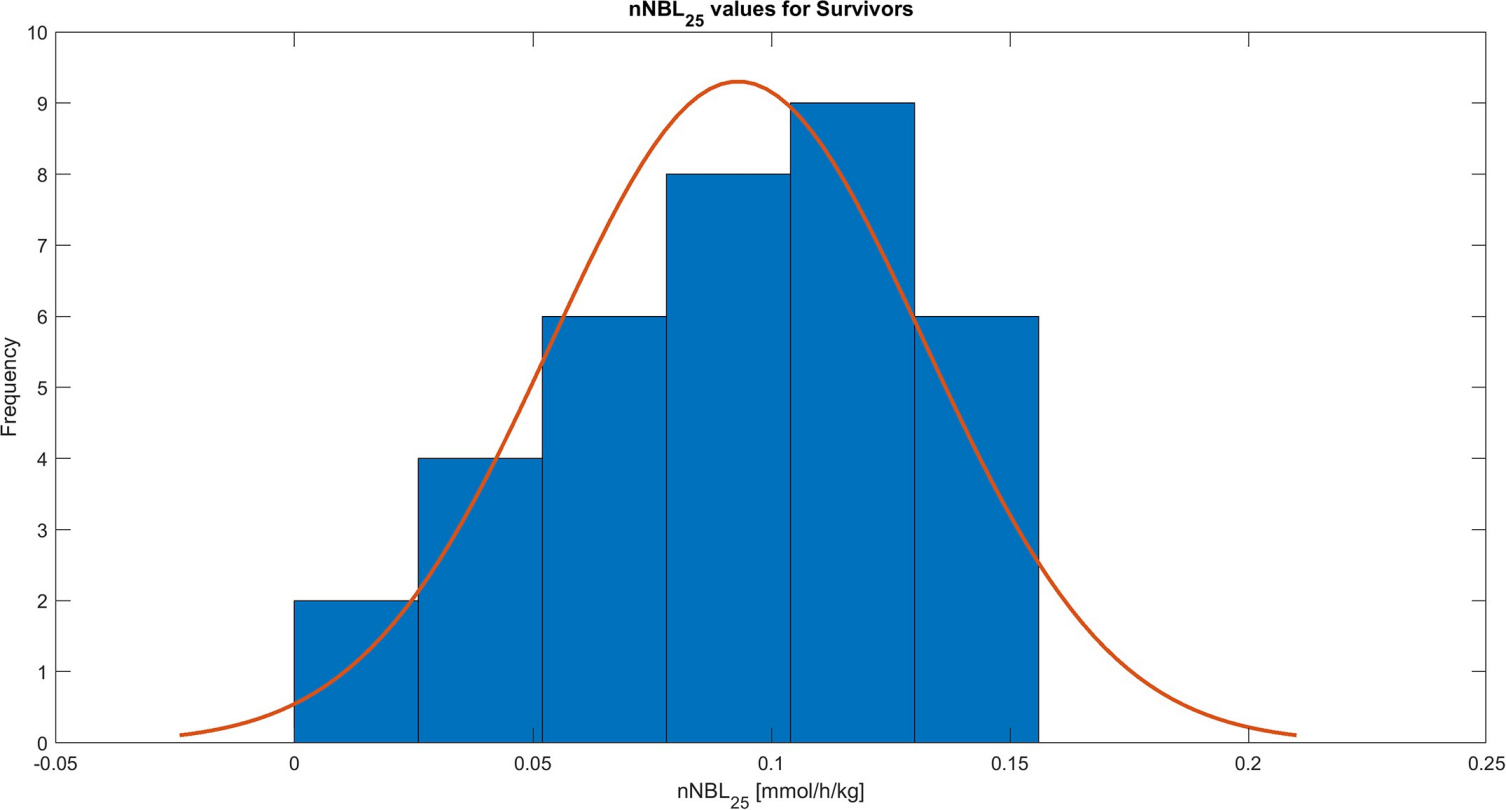
Histogram of the patient nNBL_25_ treatment means for 35 survivors, who where alive at discharge from hospital.

**Fig 3:**
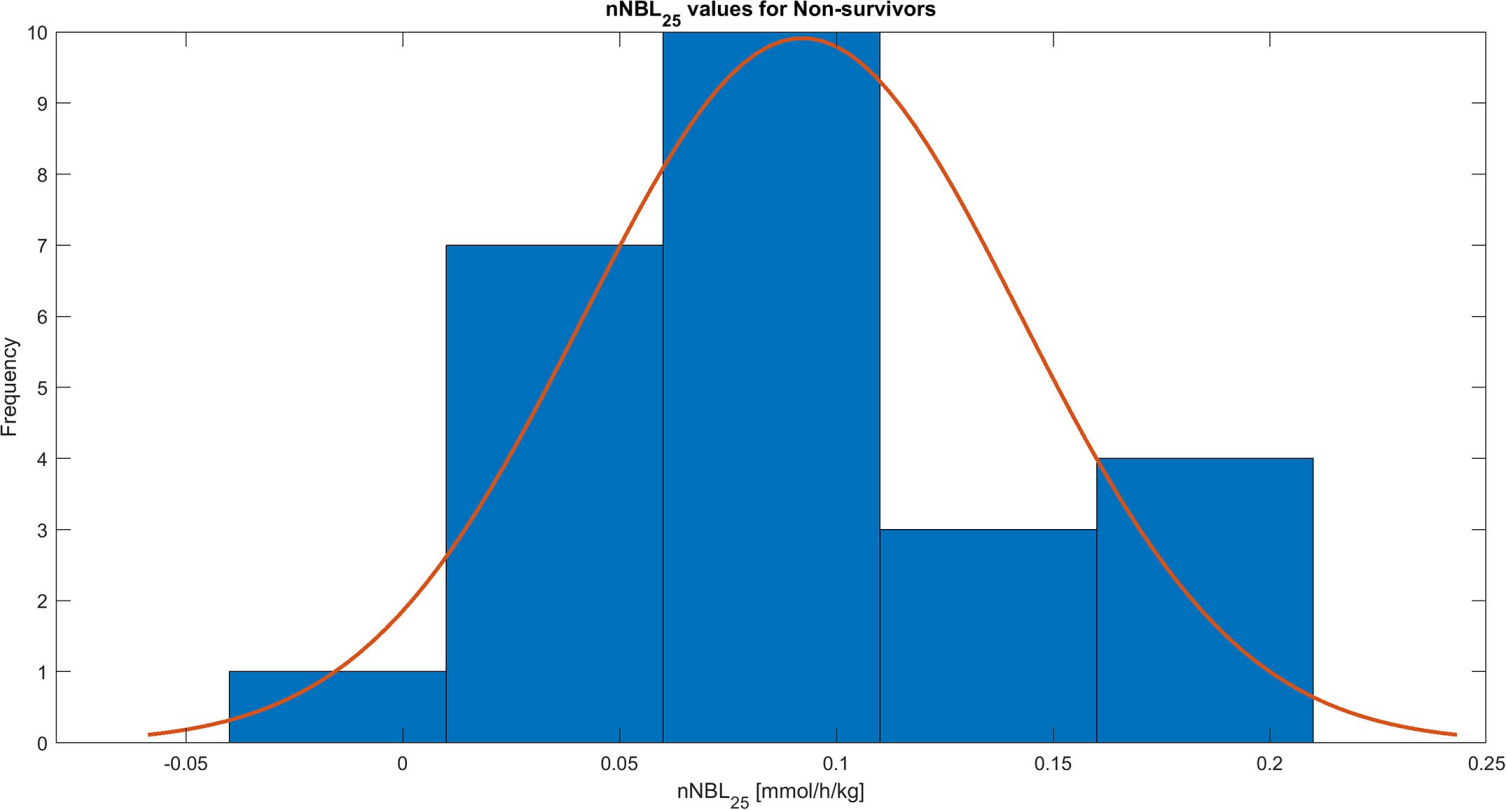
Histogram of the patient nNBL_25_ treatment means for 25 non-survivors, who died in the hospital.

During the study period of the first 72 hours of CRRT treatment the mean of all the nNBL_25_ values was 0.09 ±0.04 mmol/h/kg, calculated from records with resolution 1 minute. The distribution of the obtained nNBL_25_ values for all patients is shown in Fig 1, for survivors in Fig 2, and for non-survivors in Fig 3. The nNBL_25_ values between survivors and non-survivors did not differ significantly, p = 0.9048.

### Suggestion of how to respond to the nNBL_25_ level

**Fig 4:**
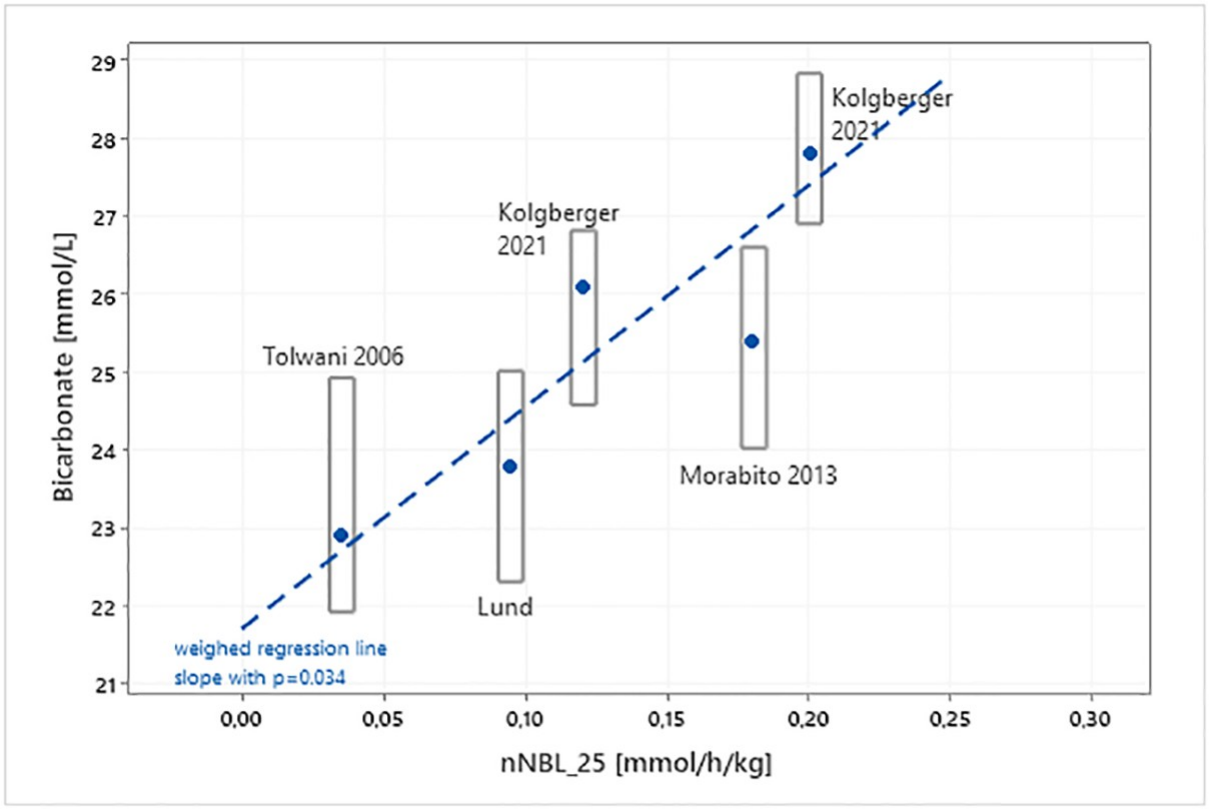
Relation between nNBL_25_ and steady state bicarbonate (HCO3-) for the actual cohort in this study (Lund) compared to previously conducted studies (Tolwani et al, Koglberger et al, Morabito et al [10–12]). nNBL_25_ was calculated from the dataset provided in each previous study for comparison to the actual cohort in this study. A regression line is shown, and the p-value is 0.034.

Based on the nNBL_25_ values in our cohort, and on the nNBL_25_ values in three historic study cohorts from literature (Fig 4), we suggest normal range and cutoff values for interpreting the nNBL_25_ output according to Table 2, including subsequent suggestions for actions [10–12].

**Table 2:** The authors’ suggestions on normal range for nNBL_25_ and high/low deviations, including suggestions how to manage a nNBL_25_ deviation.

Normalized net buffer load (nNBL_25_) [mmol/h/kg]	Status of patient and CRRT	Suggested actions
**< 0 mmol/h/kg**	Predicted net removal of HCO_3_ by the CRRT therapy. **Moving towards metabolic acidosis**.	Revise the prescription. Increase the Q_B_/Q_D_ ratio. Consider to increase citrate delivery to the circuit. Increase the CRRT treatment dose.
**0.1–0.2 mmol/h/kg**	Predicted slight net infusion of HCO_3_ of the CRRT treatment. A desired slight positive net infusion (when patient is at 25 mmol/l) exists, which balances proton generation rate from patient’s metabolism. **The acid base balance will be stable**.	No changes needed. Watchful monitoring.
**> 0.3 mmol/h/kg**	Predicted positive net HCO_3_ infusion surpassing the proton generation of the CRRT treatment. **Moving towards alkalosis**.	Revise the prescription. Decrease the Q_B_/Q_D_ ratio. Consider to decrease the citrate delivery to the circuit. Decrease the CRRT treatment dose.

It is important to understand that nNBL_25_ is calculated from parameters from the CRRT machine settings, the treatment fluid contents and the filter properties. The nNBL_25_ will predict a likely systemic impact of the CRRT circuit, and thus act as an alert to the operator.

Negative nNBL_25_ predicts net removal of HCO_3_ from the patient by the CRRT circuit. The patient will shift towards acidosis. Suggested actions are to revise the prescriptions, and to *for instance* increase the Q_B_/Q_D_ ratio, and to increase the citrate dose D_cit_, and to increase the CRRT dose.

nNBL_25_ within the normal range +0.1–0.2 mmol/L predicts a small systemic net infusion of HCO_3_ by the CRRT circuit. This balances the metabolic acidosis that always is present in a patient with AKI. The proton generation rate from patient’s metabolism is also balanced. No action is needed, except watchful monitoring.

nNBL_25_ at level above +0.3 mmol/L predicts positive net HCO_3_ infusion surpassing the proton generation of the CRRT treatment. The patient will shift towards alkalosis. Suggested actions are to revise the prescriptions, and to *for instance* decrease the Q_B_/Q_D_ ratio, and to decrease the citrate delivery D_cit_, and to decrease the CRRT dose.

### nNBL_25_ relation to the patient homeostasis

**Fig 5:**
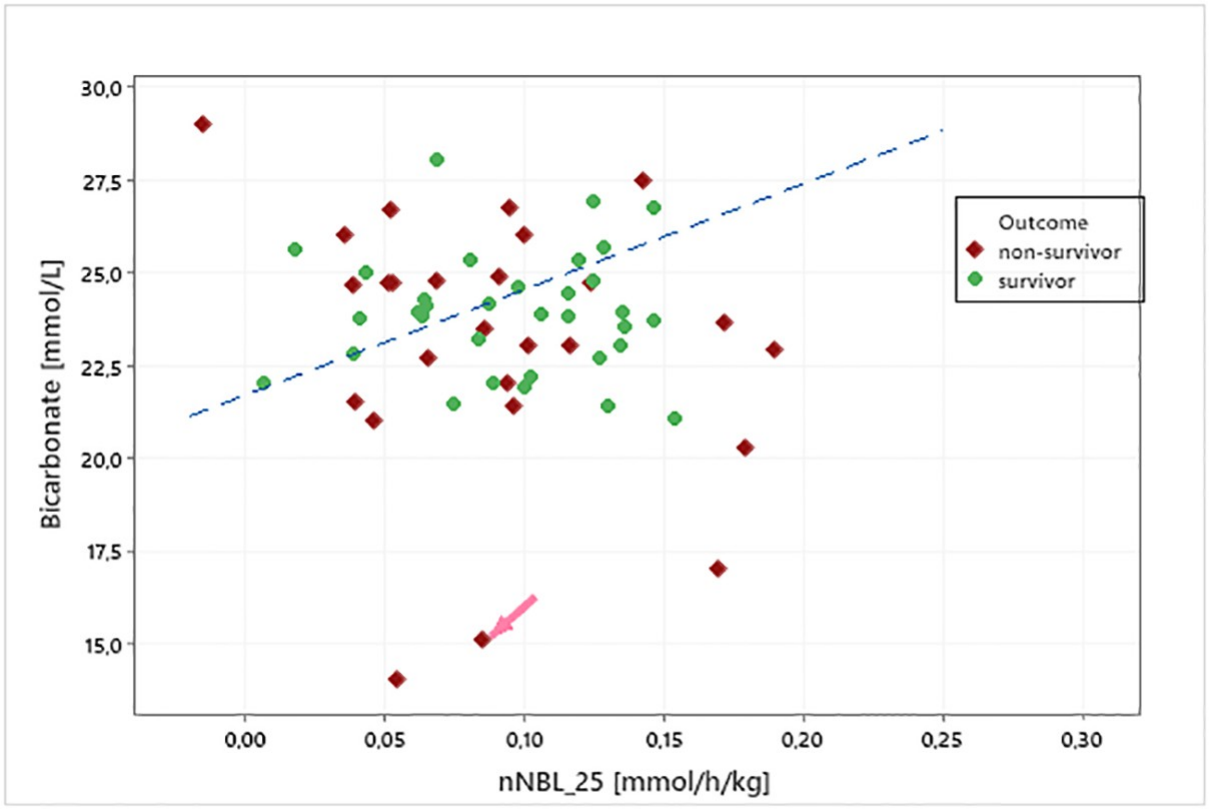
Steady state bicarbonate versus nNBL_25_ in the 60 included patients. Green dots represent survivors and red dots represents non-survivors regarding hospital mortality. The non-survivor patient marked with an arrow is further described in Fig 4.

In the present cohort we could not show a correlation between nNBL_25_ and steady state bicarbonate (Fig 5). The nNBL_25_ level in the present cohort was comparable to nNBL_25_ levels, which were calculated in three historic cohorts from literature (Fig 4).

**Fig 6:**
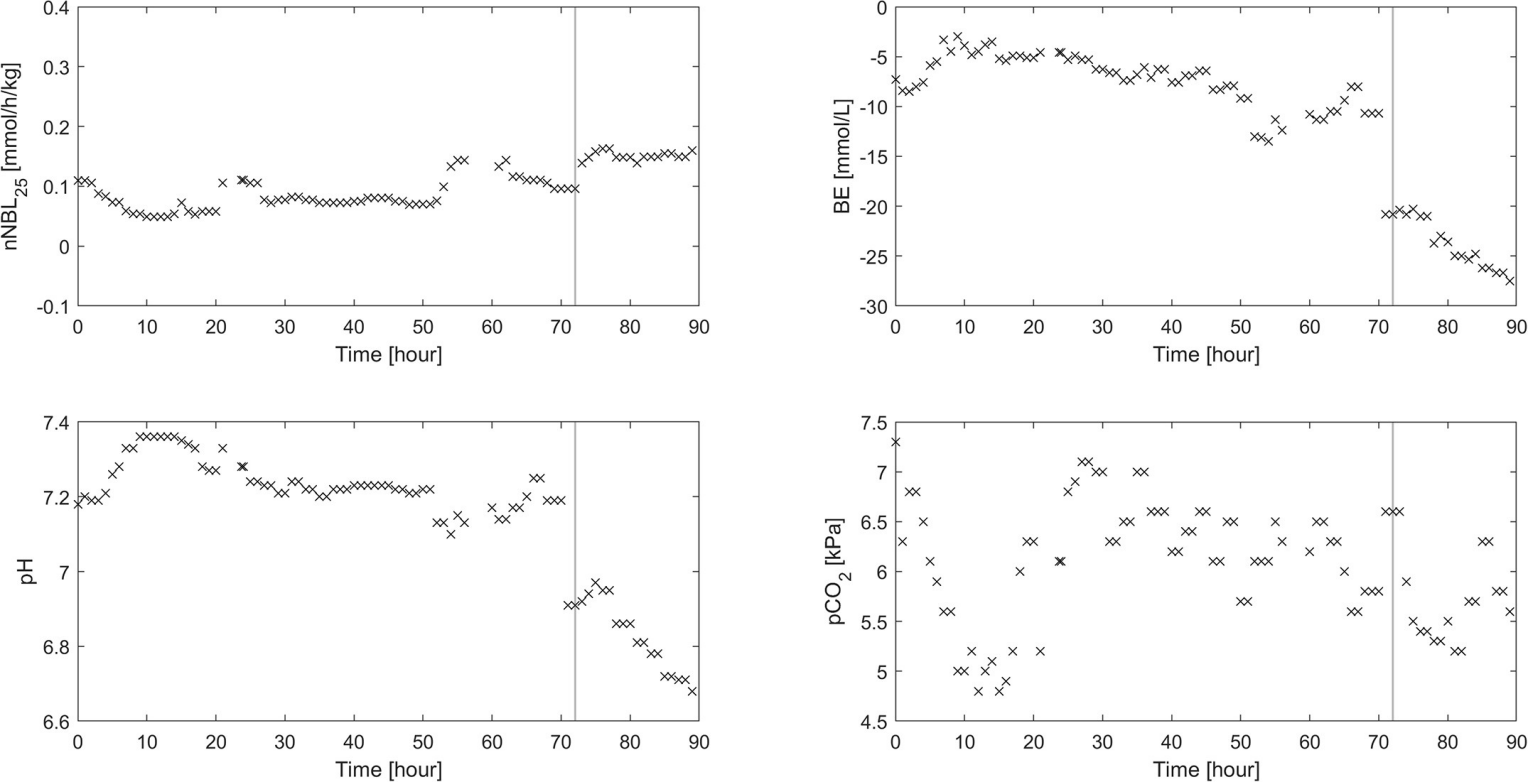
Example of a non-survivor patient with developing fatal therapy resistant shock at CRRT-hour 60 because of heart failure and subsequent fatal cardiac arrest, where pH and base excess deteriorate. nNBL_25_ is reflecting strictly the CRRT circuit’s impact on the patient.

An individual patient who deteriorated in severe shock during CRRT is shown in Fig 6, as an outlier. The patient presented massively developing negative base excess and metabolic acidosis because of heart failure and in the end fatal cardiac arrest. nNBL_25_ will strictly reflect the CRRT circuit’s impact on the unstable steady state in this patient, and cannot explain other parallel processes.

### nNBL_25_ relation to the CRRT circuit

As is apparent from the set of Eqs 1, 2 and 3, the nNBL_25_ will be dependent on many elements in the CRRT circuit. However, the element with the highest impact is the citrate containing pre-blood pump (PBP) replacement fluid infusion rate (Q_PBP_). The r^2^ Pearson correlation coefficient between the prescribed PBP replacement fluid (Q_PBP_) flow rate and the computed values of nNBL_25_ during the 60 CRRT treatments in the cohort was 0.74 for NBL_25_ not normalized to body weight (p<0.001) and 0.65 for nNBL_25_ normalized to body weight (p<0.001).

## Discussion

In this study we introduced the normalized net buffer load (nNBL_25_) parameter and computed it during 60 CRRT treatments for the first time. The nNBL_25_ will also be included in the PrisMax V3 software version (Baxter, Deerfield, USA).

The calculated nNBL_25_ estimates the steady state buffer balance between the system blood compartment and the CRRT circuit, considering both the citrate load into the patient and the bicarbonate net infusion from the bicarbonate content in the treatment fluids. In this version 1 model a fixed venous bicarbonate level of 25 mmol/L is assumed in the patient.

Review of published data available and the theoretical concept in our set of equations together with clinical circumstances in a patient with AKI, revealed that the most optimal and logical normal range of the nNBL_25_ is 0.1–0.2 mmol/h/kg, i.e. a slight positive net bicarbonate balance opposing the metabolic acidosis originating from the AKI state in patients in need of CRRT. nNBL_25_ values > 0.3 mmol/h/kg imply a risk of developing metabolic alkalosis, whereas negative values imply a risk of developing metabolic acidosis (Figs 1–3, Table 2) [10–12].

A negative nNBL_25_ means that the CRRT circuit will remove bicarbonate from the patient’s blood compartment over the filter and thus create an acidifying effect in the patient. To correct this the Q_B_/Q_D_ ratio can be increased in order to remove less bicarbonate. Another option would be to use treatment fluids with higher bicarbonate concentration.

The optimal nNBL_25_ is slightly positive, i.e. the circuit will deliver a small amount of bicarbonate to the patient circuit. This is beneficial for the patient with AKI and an existing adjacent metabolic acidosis.

However, too much positive nNBL_25_ means that the circuit delivers excess amount of bicarbonate to the patient and creates an alkalizing effect. In order to correct this situation the Q_B_/Q_D_ ratio can be decreased so that the circuit will deliver less bicarbonate to the patient blood. Another option would be to use treatment fluids with lower bicarbonate concentration.

However, real life is even more complex; the bicarbonate balance between the CRRT circuit and the patient blood compartment will generate an impact that comes on top of an existing complicated acid balance disturbance in a critically ill patient.

The nNBL_25_ value is an estimate of what can be expected with the given treatment fluids (with their bicarbonate contents) in use, the properties of the filter membrane, and the fluid flows. Each time the fluid flows are changed the nNBL_25_ the will be recalculated and get a new value, which is presented on the CRRT machine interface.

Clinically it is important to understand how nNBL_25_ is computed from 1. the CRRT machine fluid flow settings, 2. the treatment fluids used, and 3. the properties of the filter used, and that it is an estimated parameter. Thus, the nNBL_25_ version tested in this study can not consider parallel administration of citrate for instance from blood transfusions, nor parallel administration of bicarbonate. This may lead to a systemic underestimation of nNBL_25_ and hence the actual buffer load during CRRT.

Regarding outliers, nNBL_25_ values did not always correlate strictly to the acid-base status of the patient as shown in Fig 6. In this case the patient suffered a cardiac arrest and passed away. The deteriorating catastrophe imposed a massive metabolic acidosis changing the conditions of the steady state with a huge proton generation. This particular patient constitutes an example showing that underlying clinical problems can interfere with nNBL_25_. The top underlying problems affecting the steady state compulsory for computing the nNBL_25_ to steady state can be severe septic shock, cardiac arrest, ARDS, and high blood ketones and lactate. In a critically ill patient, the acid-base status is complex and multifactorial, and the impact of the CRRT circuit comes on top of existing disturbances, a phenomenon also described by Lee et al. [13]. The outliers were often seen in non-surviving patients, where CRRT could not correct the acid-base disturbances, and these patients showed deranged and rapidly deteriorating underlying acid-base statuses (Fig 6).

The net infusion of buffer is expected to neutralize the proton (H^+^) generation rate G_H+_ from the metabolism. According to literature, G_H+_ is 0.04 mmol/h/kg for an average human. The production of protons from the metabolism is strongly dependent on the patient´s protein catabolism, and it is 2–3 times less than the optimal nNBL_25_ steady state of 0.1–0.2 mmol/h/kg as suggested in Table 2 and Figs 1–3. Critically ill patients most probably will have a higher proton generation rate, and a greater variability [14,15]. Equality between proton generation rate in the body G_H+_ and net buffer load nNBL_25_ defines the steady state. Arterial blood samples are used in this study, while the net buffer load equations are based on venous samples. There might be a slight difference of 1 mmol/L in bicarbonate level between arterial (lower) and venous blood [16]. If nNBL_25_ surpasses the proton generation rate (nNBL_25_ > G_H+_), steady state equilibrium above venous HCO_3_^-^ 25 mmol/L will occur and thus result in alkalosis. If nNBL_25_ < G_H+_ the patient will become acidotic since the buffer infusion cannot compensate for the G_H+_ at steady state [17].

The range of nNBL_25_ was relatively narrow in the study cohort due to the strict prescription protocol in use at the Adult Intensive Care Unit, Skåne University Hospital, Lund, Sweden, compared with nNBL_25_ values calculated from the historic cohorts. Koglberger et al. studied the buffering effect of Phoxilium versus Biphozyl replacement fluids and could note that different bicarbonate levels affected the acid base status in the patient [10]. Morabito et al. described the effect of changing to treatment solutions with higher citrate content. A better acid-base control without clinically relevant alkalosis could be seen [11]. Tolwani et. al. investigated the metabolic effects of two different citrate solutions infused at the same flow rate and could discover that higher citrate concentration was connected to mild metabolic alkalosis [12].

Metabolic acidosis is the most common acid-base disorder occurring in almost 80% of the admitted ICU patients [18]. A majority of the patients need invasive respiratory support, which corrects respiratory deviations, but at the same time makes patient-driven respiratory compensation impossible, due to the preset minute volume in the ventilator. For some patients, worsening acid-base parameters could be seen during the 72-hour study period regardless of CRRT treatment, thus the acid-base deviation could not be corrected, a phenomenon discussed by Lee et. al [13]. It was concluded that unfavorable metabolic acidosis trajectory determines worse outcome. In our cohort, we could see a signal that the group of patients that diverged most from the correlation between steady-state acid-base status and nNBL_25_ were the non-survivors (Fig 4). Deranged liver function can have an impact on the metabolic capacity of citrate and can thus change the preconditions for reaching steady state. Also, prolonged CRRT imposes high risk of metabolic alkalosis, as does recovery from severe lactic acidosis. This study did not cover real life scenarios after the study period, which is a limitation.

There were several discrepancies in the raw data, such as missing data and wrong data. Since the study was retrospective, it was in some circumstances difficult to understand the underlying reasoning of the adjustments of the CRRT treatment settings. It was also in some instances difficult to find clear correlations between the treatment setting changes and the underlying physiological parameters. Also, the cohort of 60 patients is rather small.

## Conclusion

It was possible to calculate realistic nNBL_25_ values in 60 CRRT treatments in our retrospective observational cohort by using our set of equations for the net buffer load (nNBL_25_) version 1. The nNBL_25_ parameter is to be considered as a safety parameter during citrate anticoagulated CRRT. It can alert the operator about the impact of the CRRT circuit on the patient’s systemic acid base status by taking into account systemic bicarbonate generated from citrate metabolism and infused bicarbonate from the treatment fluids. In our cohort the nNBL_25_ levels were comparable to nNBL_25_ levels calculated in historic cohorts from literature.

## Supporting information

S1 File (DOCX)
