# Supplementary material for: Net buffer load during regional citrate anticoagulated continuous renal replacement therapy
Source: PLoS One. 2025 Jan 30;20(1):e0315727. doi: 10.1371/journal.pone.0315727 (PMC11781738; doi:10.1371/journal.pone.0315727)
Supplement: S1 File — (DOCX) [file pone.0315727.s001.docx]

The Flexitrate protocol.

|  | **High PF-Ca2+**  **>0.50 mmol/l** | **Normal PF-Ca2+**  **0.25-0.50 mmol/l** | **Low PF-Ca2+**  **<0.25mol/l** |
| --- | --- | --- | --- |
| **Low**  **patient Ca2+**  **<1.0 mmol/l** | Increase citrate  dose by 0.5 mmol/l  and calcium  infusion by 5-10% | Increase  calcium infusion  by 5-10% | Decrease  citrate dose  by 0.5 mmol/l |
| **Normal**  **patient Ca2+**  **1.0-1.2 mmol/l** | Increase citrate  dose by 0.5 mmol/l | No change | Decrease  citrate dose  by 0.5 mmol/l |
| **High**  **patient Ca2+**  **>1.2 mmol/l** | Decrease calcium  infusion by 5-10% | Decrease  calcium infusion  by 5-10% | Decrease  citrate dose  by 0.5 mmol/l  and calcium  infusion by  5-10% |
